# A Platform for Testing the Biocompatibility of Implants: Silicone Induces a Proinflammatory Response in a 3D Skin Equivalent

**DOI:** 10.3390/biomedicines12010224

**Published:** 2024-01-19

**Authors:** Rima Nuwayhid, Torsten Schulz, Frank Siemers, Jeannine Schreiter, Philipp Kobbe, Gunther Hofmann, Stefan Langer, Olga Kurow

**Affiliations:** 1 Department of Orthopaedic, Trauma and Plastic Surgery, University Hospital Leipzig, 04103 Leipzig, Germany; torsten.schulz@medizin.uni-leipzig.de (T.S.); stefan.langer@medizin.uni-leipzig.de (S.L.); 2 Department of Plastic, Hand Surgery and Burn Care, BG Klinikum Bergmannstrost, 06112 Halle, Germany; frank.siemers@bergmannstrost.de; 3 Klinik am Rosental GmbH, 04105 Leipzig, Germany; jeannine.schreiter@klinik-am-rosental.de; 4 Department of Trauma and Reconstructive Surgery, Martin-Luther-University Halle-Wittenberg, 06120 Halle, Germany; philipp.kobbe@bergmannstrost.de; 5 Department of Trauma and Reconstructive Surgery, BG Klinikum Bergmannstrost, 06112 Halle, Germany; 6 Department of Trauma, Plastic and Reconstructive Surgery, University Hospital Jena, 07747 Jena, Germany; gunther.hofmann@med.uni-jena.de

**Keywords:** tissue engineering, 3D skin equivalent, 3D skin model, silicone, breast implants, biocompatibility, cytotoxicity, cytokine response, Swanson prosthesis, silicone prosthesis

## Abstract

Biocompatibility testing of materials is carried out in 2D cell cultures or animal models despite serious limitations. 3D skin equivalents are advanced in vitro models for human skin. Silicone has been shown to be noncytotoxic but capable of eliciting an immune response. Our aim was to (1) establish a 3D skin equivalent to (2) assess the proinflammatory properties of silicone. We developed a coculture of keratinocytes and fibroblasts resulting in a 3D skin equivalent with an implant using samples from a breast implant. Samples with and without the silicone implant were studied histologically and immunohistochemically in comparison to native human skin samples. Cytotoxicity was assessed via LDH-assay, and cytokine response was assessed via ELISA. Histologically, our 3D skin equivalents had a four-layered epidermal and a dermal component. The presence of tight junctions was demonstrated in immunofluorescence. The only difference in 3D skin equivalents with implants was an epidermal thinning. Implanting the silicone samples did not cause more cell death, however, an inflammatory cytokine response was triggered. We were able to establish an organotypical 3D skin equivalent with an implant, which can be utilised for studies on biocompatibility of materials. This first integration of silicone into a 3D skin equivalent confirmed previous findings on silicone being non-cell-toxic but capable of exerting a proinflammatory effect.

## 1. Introduction

In accordance with the precept primum non nocere, biocompatibility might be the most important quality of every implant inserted into the body. Thus, extensive research on interactions between implants and their surrounding tissues is required.

This is often performed in animal models despite their numerous disadvantages: they are expensive and highly regulated in many countries and thus laborious; have relevant moral implications associated with them; and most importantly, are an inadequate representation of human physiology, producing misleading results [1,2,3]. Therefore, in vitro cell culture models are required for adequate investigations on biocompatibility.

Three-dimensional (3D) tissue equivalents have proven to be superior to 2D cell cultures for in vitro studies, as they can be composed of multiple cell types with control over cell type composition [4,5]. The cells in 3D tissue equivalents are able to differentiate and form an organotypical architecture [6]. They provide enhanced cell–cell contacts, and as an extracellular matrix is present, they also contain cell–matrix contacts [4,6,7,8,9,10]. This is especially relevant in modelling skin with its distinct layered build-up and multitudinous cell contacts to fulfil its barrier functions. Thus, 3D skin equivalents have been used in a variety of studies, e.g., to assess skin infections, the effect of cancer therapeutics, and the toxicity or antibiofilm properties of various agents [6,8,11,12,13].

Silicone was first implanted into the human body as a device for bile duct repair in 1946 by Lahey [14]. Since then, its stability, elasticity and reported cytocompatibility made it a regularly used component of implants for different indications. Silicone rods have been used in hand surgery for staged flexor tendon repair for decades [15]. Swanson prosthesis for proximal interphalangeal and metacarpophalangeal joint arthroplasty are made entirely out of silicone [16]. Apart from usage in degenerative diseases such as rheumatoid arthritis and post-traumatic arthrosis, they have also been described as replacements in acute hand injuries [17]. Perhaps the most widely known utilisation of silicone implants is breast reconstruction. One common denominator in these constellations is the direct contact of the silicone implant with skin: the digits physiologically contain very little subcutaneous tissue, which is further reduced during preparation or during debridement in case of infections. During nipple- or skin-sparing mastectomy, either for surgically treating breast cancer or risk-reducing procedures, the subcutaneous tissue is thinned out as much as possible in order to minimise recurrence rates before a silicone implant is placed for breast reconstruction [18]. Other applications of silicone include ophthalmology, maxillofacial and nasal reconstruction, and various devices, such as urinary catheters, venous port catheters, or drivelines of ventricular assist devices [19,20,21,22,23,24]

While at first silicone as an entirely synthetic material was reported to be immunologically inert, histological studies in animals in the 1980s demonstrated an immunologic effect [25]. The cytotoxicity and inflammatory activity of silicone have been studied in vitro and in vivo using murine models [26]. In vitro studies on different cell types (macrophages, monocytes, fibroblasts, umbilical vein endothelial cells) confronted with silicone have been performed [27]. Keratinocytes, which play an important role as an immunologic barrier, have not yet been tested in terms of their reaction to silicone [27,28].

Biocompatibility testing of medical devices is regulated by International Standard ISO 10993 [29]. For devices to be categorized as risk-adapted, in vitro studies in 2D cell cultures and in vivo studies in animal models may be required. Considering the aforementioned limitations of both test systems, a 3D in vitro test model using human cells would be highly useful, especially in light of variations of silicone and combinations with different coatings being introduced.

3D skin equivalents have previously been used to assess the skin integration of percutaneous devices [23,30]. Mock drivelines have been manufactured from poly-ε-caprolactone, seeded with fibroblasts, and inserted into the 3D skin equivalents through biopsy punch holes, thus mimicking transepidermal implants. However, data on intra- or subdermal implants with no direct contact to keratinocytes are still lacking as are data on the interactions of silicone implants with keratinocytes.

Our group previously established an organotypic 3D equivalent of human pleura and successfully mimicked pleural empyema [31,32]. The aim of the present study was to establish a 3D skin equivalent, which would allow for investigations of interactions between human skin cells (keratinocytes and fibroblasts) and implants of different materials, starting with silicone, to assess biocompatibility.

## 2. Materials and Methods

### 2.1. Composition of 3D Skin and Implanted 3D Skin Equivalents

**Figure 1:**
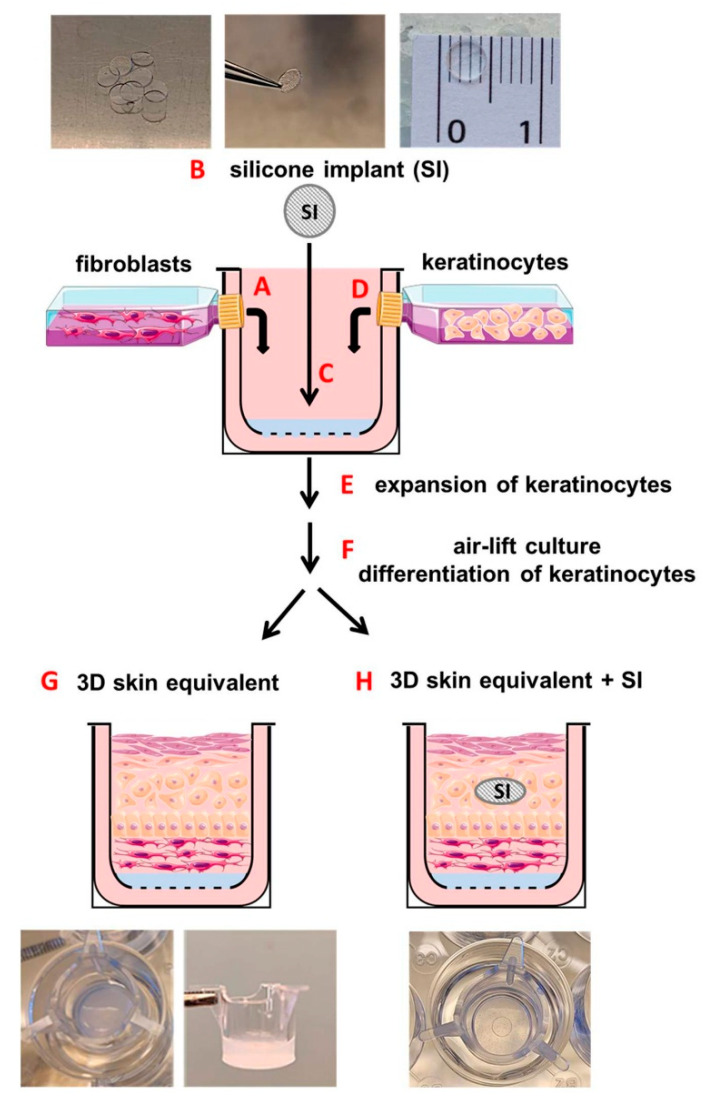
Schematic overview of experimental methods for construction of the 3D skin equivalent and 3D skin equivalent with an implant using a three-dimensional collagen gel culture. A single cell suspension of human fibroblasts (HDFp) obtained from confluent monolayers was passaged and cultivated in a type I collagen gel matrix (HDFp-collagen gel) (**A**). Silicone samples were cut out using a biopsy punch (**B**) and implanted in HDFp–collagen gel constructs (**C**). After solidification of HDFp–collagen gel constructs, keratinocytes were placed on the gel matrix to build up a three-dimensional skin-like structure (**D**). The skin constructs were cultured for another 3 days for keratinocytes to expand (**E**). The following air-lift culturing technique (**F**) allowed for cell differentiation. Eventually, the 3D equivalent had a skin-specific stratification and morphology (**G**,**H**).

Our 3D skin equivalent is shown schematically in Figure 1. It was based on fibroblasts (HDFp, primary human dermal fibroblasts, CELLnTEC Advanced Cell Systems AG, Bern, Switzerland), supplemented by human epidermal keratinocyte cells (HDEK, human epidermal keratinocytes, CELLnTEC). Collagen gel was prepared using rat-tail type-I collagen (ibidi GmbH, Gräfelfing, Germany) to a final concentration of 5 mg/mL in DMEM, neutralized with 5 M NaOH.

Collagen gel (500 μL) with fibroblasts (HDFp-collagen gel) at a final cell density of 3.5 × 10^4^ cells/mL was pipetted into 12-well inserts with a polymer mesh (ThinCert, 8 μM pore size, translucent, Greiner Bio-One GmbH, Frickenhausen, Germany). These inserts were then positioned into 12-well plates (Figure 1A).

In order to produce a 3D skin equivalent with an intradermal implant, we added silicone samples to half of our cell cultures. The implant samples were obtained from the envelope of a commercially available breast implant with a smooth surface. Using a biopsy punch, we cut out circular samples with a diameter of 5 mm and thickness of 0.4 mm and sterilised and bathed them in ethanol and sterile phosphate-buffered saline (Dulbecco’s PBS, Gibco, Themo Fisher Scientific, Waltham, MA, USA). Our target specifically was the intradermal positioning of the implants. In order to keep them from sinking to the bottom of the transwell, where they would have no tissue contact on their basal side, we first placed the HDFp-collagen gel in the transwell. We awaited spontaneous polymerisation for 10 min and then placed the silicone samples on top. With the collagen gel having partially polymerised, the implants only sank about half way into the desired position. (Figure 1B). These 3D constructs were then polymerized at 37 °C in 5% CO_2_ for 5 h and then equilibrated in fibroblast growth medium CnT-PR-F (CELLnTEC) overnight.

After 24 h of polymerisation of the HDFp-collagen gel, the 3D constructs were coated with 100 μL of fibronectin (5 mg/mL) (Sigma-Aldrich, St. Louis, MO, USA) in DMEM without FBS and incubated at 37 °C for 1 h. After that, organotypic 3D skin equivalents were generated by seeding 3.2 × 10^5^ keratinocytes suspended in 250 µL of CnT-PR-medium (Cellntec, Bern, Switzerland) on top of the collagen gel in each transwell (Figure 1D). Keratinocyte’s adhesion to the collagen was allowed for 5 h by incubating the transwells at 37 °C in 5% CO_2_ without medium. Five hours after keratinocyte seeding, we applied 1 mL of CnT-PR-medium outside and 500 µL inside of the ThinCert. These inserts were then cultured in keratinocyte growth medium for 72 h until the keratinocytes expanded and covered the entire surface of the insert (Figure 1E). At this point, the inserts were lifted to an air–liquid interface (Figure 1F) with 1 mL of CnT-PR-FTAL-medium (Cellntec) outside of the ThinCert to ensure exposure of keratinocytes to air. 3D cocultures were cultured for an additional 26 days with the 3D medium being replaced every three days. All experiments were performed on 30-day cultures. Under the influence of air and an increased Ca^2+^ concentration in the medium, this 3D coculture differentiated into an organotypic 3D human skin equivalent (Figure 1G) or 3D human skin equivalent with the intradermal implant (Figure 1H).

### 2.2. Histochemistry and Immunofluorescence

We compared our 3D skin equivalent to human skin tissue using light microscopy and immunofluorescence. Human skin was obtained from patients undergoing abdominoplasty at the Department of Plastic Surgery, University Hospital Leipzig, Leipzig, Germany, (approved by the Ethics Committee of the University of Leipzig, Leipzig, Germany (434/20-ek)).

Samples were placed in zinc-formaldehyde (Sigma Aldrich, Taufkirchen, Germany) directly after being harvested in the operation theatre, fixed at 4 °C for 24 h, then paraffin-embedded and microtome-cut onto Superfrost Plus-charged slices (Themo Fisher Scientific, Waltham, MA, USA), then deparaffinized in xylene, and hydrated with alcohol prior to standard hematoxylin and eosin staining. For immunohistochemical experiments, the peroxidase was inactivated with 1% H_2_O_2_ in methanol for 10 min. Slides were boiled in 0.01 M sodium citrate with pH 6.0 for 10 min to retrieve antigens. Unspecific binding site saturation was consistently carried out with 10% goat serum in 0.1 M PBS with pH 7.4 (130 mM NaCl, 7 mM Na_2_HPO_4_, 3.5 mM NaH_2_PO_4_, 7.7 mM NaN_3_, 0.1% BSA, 0.2% Triton X-100, 0.05% Tween 20 and 10% serum of host of secondary antibody; 1 h at RT). Samples were incubated overnight at 4 °C with primary antibodies against filaggrin, involucrin, cytokeratin 1, keratin 17, E-Cadherin, and ZO-1 diluted in blocking buffer. After three washes in TBS for 10 min, slides were incubated with goat anti-rabbit Alexa Fluor-labelled secondary antibodies diluted in blocking buffer (Table 1). After another three washes in TBS for 10 min, cover ships were mounted with media containing DAPI (Vector Laboratories, Inc., Newark, CA, USA). Appropriate negative controls for the immunostaining were prepared by omitting the primary antibody. Localization of staining was analysed via fluorescent microscopy with a Leica Axiovert 100 microscope equipped with a Leica digital camera.

### 2.3. 3D Skin Equivalent Response Quantification

In a first step, we assessed the cytotoxicity induced by the silicone implants. Culture supernatants below the inserts of 3D skin equivalents with implants were harvested at days 5, 10, 15, 20, 25, and 30 after 3D skin equivalent production, and the cytotoxicity of silicone was measured via lactate dehydrogenase assay with a Cytotoxicity Detection Kit (Merck KGaA, Darmstadt, Germany) according to the manufacturer’s instructions. Samples of 3D skin equivalent without implants or samples of 3D skin equivalent without implants treated with 5% Triton X-100 were used as the negative (0% toxicity) and positive (100% toxicity) control, respectively.

In a second step, we quantified the levels of inflammation mediators. Concentrations of IL-1α, IL-6, IL-8, IL-33, MCP-1, and TNF-α were measured in the culture supernatants of our 3D skin equivalent without the implant and in the 3D skin equivalent with the silicone implant using ELISA kits (R&D Systems, Inc., Minneapolis, MN, USA) on day 30 of cultures.

## 3. Results

### 3.1. Establishment of 3D Skin Equivalents with and without Silicone Implants

**Figure 2:**
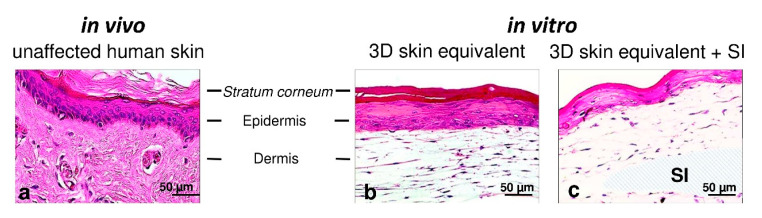
Representative photomicrographs of unaffected human skin and the 3D skin equivalent with and without the silicone implant. H&E staining showing unaffected human skin (**a**) and the 30-day 3D skin equivalent (**b**) and the 3D skin equivalent with the silicone implant (**c**) at 20× magnification. SI—silicone implant.

**Figure 3:**
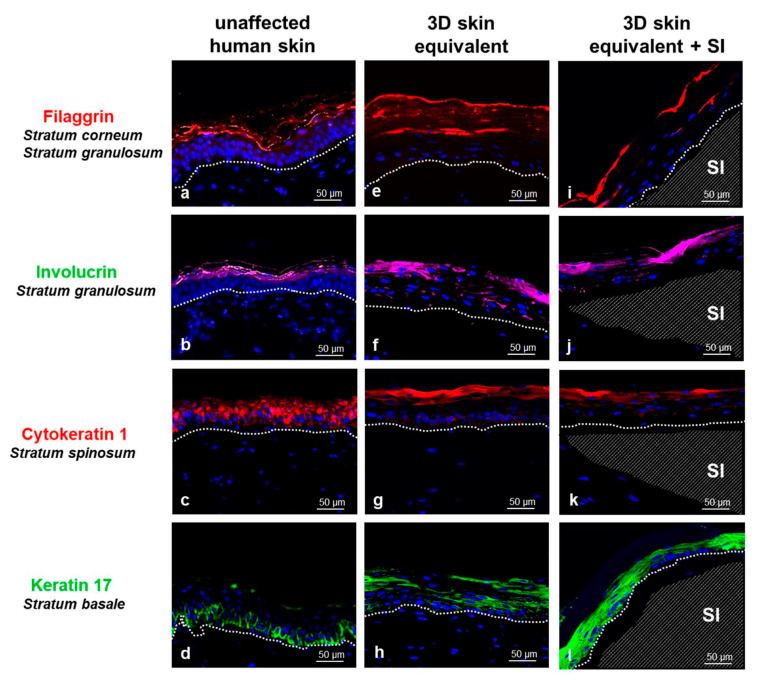
Comparison of unaffected human skin (**a**–**d**) with our 3D skin equivalent with (**i**–**l**) and without implant (**e**–**h**) by immunofluorescence staining (20×). The visible distinction between epidermis and dermis is marked with a dashed line (**a**–**l**), the position of the silicone implant (SI) with a shaded area (**i**–**l**). Immunostaining demonstrates the expression of filaggrin in red (**a**,**e**,**i**), involucrin in green (**b**,**f**,**j**), cytokeratin 1 in red (**c**,**g**,**k**) and keratin 17 in green (**d**,**h**,**l**) with counterstaining of cell nuclei with DAPI (blue). Controls in which the primary antibody was omitted showed no nonspecific binding. Scale bar = 50 μm.

The 3D human skin equivalent was set up with human keratinocytes and fibroblasts (Figure 1). In order to generate an in vitro model for skin with a silicone implant, fibroblasts were mixed as described in the Materials and Methods section and graphically illustrated in Figure 1A and pipetted in 12-well inserts, which were placed into 12-well plates. The silicone implants were inserted into HDFp-collagen gel as previously described (Figure 1C). 3D HDFp–collagen gel constructs were cultured at 37 °C for 24 h for complete collagen solidification and supplemented with human epidermal keratinocyte cells (Figure 1D). Under the influence of air and an increased Ca^2+^ concentration in the medium (Figure 1E,F), this organotypic 3D coculture differentiated into a 3D skin equivalent (Figure 1G) and 3D skin equivalent with an intradermal implant (Figure 1H) by forming the corresponding stratification of dermis and epidermis with the stratum corneum (Figure 2).

H&E staining confirmed that our 3D skin equivalents displayed tissue morphology close to native human skin. It was found that HPEK formed a continuous layer on the 3D fibroblast matrix (Figure 2b) as keratinocytes do in human skin in vivo (Figure 2a). While a continuous layer of keratinocytes also formed in the 3D skin equivalent with implant, the epidermal component was remarkably thinner than that in the 3D skin equivalent without implant (Figure 2c). The dermal growth patterns on the silicone implant suggests that the presence of silicone does not affect fibroblasts.

### 3.2. Validation of Layered Architecture in Newly Developed 3D Skin Equivalent

In order to conduct a direct comparison between our new organotypic 3D skin equivalent and the in vivo situation, we compared our 3D skin equivalent with and without implant to human skin tissue using light microscopy and immunofluorescence (Figure 3). The human skin is divided into two different layers: the epidermis with the corneal layer (St. corneum), St. granulosum, St. spinosum, and St. basale; the dermis; and the subcutis. Histological comparison of the 3D skin equivalents with and without implants to biopsies of human skin showed the presence of both cell types (HPEK and HDFp). The epidermal component in our 3D skin equivalents was visibly divided into four layers, demonstrating skin-like morphology. The distribution of the characteristic marker proteins for each of the four epidermal layers, which are physiologically expressed by human skin in vivo, was analysed with immunofluorescence staining. Filaggrin (St. corneum and St. granulosum markers), involucrin (St. granulosum marker), cytokeratin 1 (St. spinosum marker), and keratin 17 (St. basale marker) were expressed in the skin biopsies (Figure 3a–d) and presented an expression pattern similar to our 3D skin equivalent (Figure 3e–h) and our 3D equivalent with implant (Figure 3i–l). A comparison of the graphic abstract of our model (Figure 1) and immunostaining (Figure 3) to further demonstrate the stratification can be found in the Supplementary Materials.

While we observed a thinning of St. corneum in the 3D skin equivalents with implants, this did not lead to a change in the structure of the skin layers.

Keratin 17 was expression was notably higher in the 3D skin equivalents with implants.

### 3.3. Cell Contacts

The skin is a multifunctional organ with a central role in the mechanical and immunological protection of the body, the absorption of stimuli, the exchange of substances, and the regulation of body temperature, filtration, and absorption. Cell–cell contacts of keratinocytes are of great importance for these processes and also for wound healing and immune response. If these functions are disrupted or imbalanced by injury or genetic defects, the barrier function of the skin is impaired and skin diseases occur. Thus, without cell–cell contacts, our in vitro model would not represent human skin adequately.

**Figure 4:**
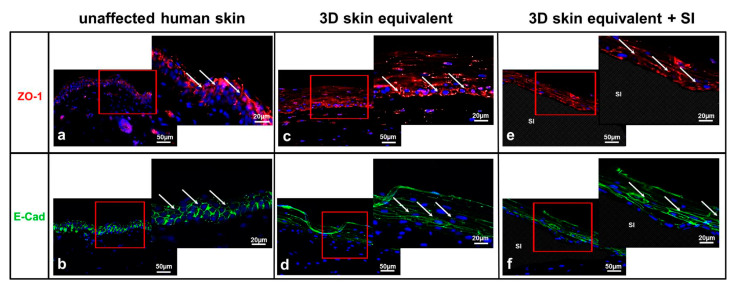
Expression of tight junctions in unaffected human skin (**a**,**b**) and the 3D skin equivalent with (**e**,**f**) and without implant (**c**,**d**). Immunofluorescence of ZO-1 (**a,c,e**) and E-Cadherin (**b,d,f**). The staining of ZO-1 (red) and E-cadherin (green) clearly showed the cell membranes of keratinocytes in the epidermis. The cell nuclei were counterstained with 4,6-diamidino-2-phenylindole (DAPI) (blue). Human fibroblasts showed no expression of ZO-1 or E-cadherin at cell membranes. The red box marks the area shown in detail to its right. Arrows signify TJ. Controls in which the primary antibody was omitted showed no specific staining. Scale bar = 50 μm.

Therefore, we investigated the localisation of epithelial cell–cell adhesion molecules (tight junction (TJ)) in the 3D skin equivalent. To determine the functional coupling and interaction of the HPEK cells with each other, we performed immunofluorescence staining of the TJ proteins E-cadherin and ZO-1 (Figure 4). The 3D skin equivalent featured intercellular tight junctions (TJ) resembling those found in human tissue (Figure 4b,c). Morphological analysis of TJ proteins showed that HPEK could polarise and form well-developed intercellular junctions with a similar expression pattern within our 3D skin equivalent (Figure 4c,d) and our 3D skin equivalent with implant (Figure 4e,f). TJ proteins were seen mainly on the surface of keratinocytes of the St. spinosum and granulosum. No difference was detected between the 3D skin equivalents with and without implant in expression and localization of epithelial cell–cell adhesion molecules.

Thus, our model allows the investigation of cell interactions by direct contact of both cell types in the 3D skin equivalent and models the physiological paracrine signal transmission better than do 2D cultures.

### 3.4. Cytotoxicity/Cytokine Response/Inflammatory Activity

In this part, we characterised the cellular responses of 3D skin equivalents to silicone by assaying the growth media underneath the cell culture inserts.

**Figure 5:**
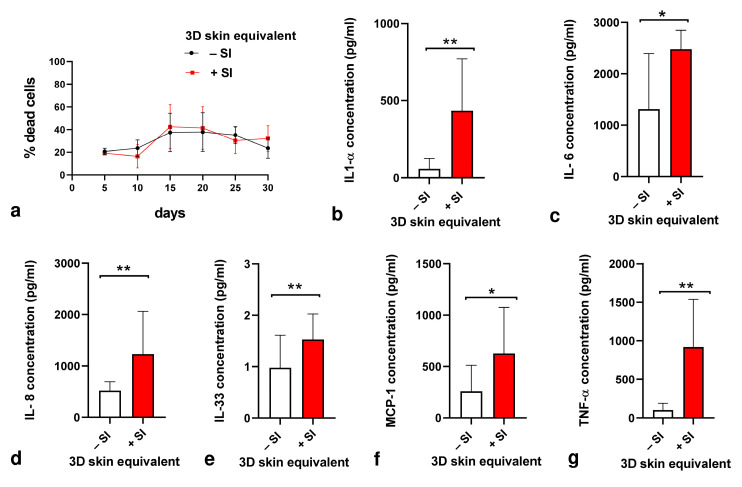
Cytotoxicity and proinflammatory cytokine production in response to silicone implantation in 3D skin equivalent. (**a**) Culture supernatants were used for measuring cytotoxicity via lactate dehydrogenase assay on days 5, 10, 15, 20, 25, and 30 after 3D skin equivalent production. (**b**–**g**) The concentrations of IL-1α (**b**), IL-6 (**c**), IL-8 (**d**), IL-33 (**e**), MCP-1 (**f**), and TNF-α (**g**) in culture supernatant were quantified with ELISA on day 30 of cultures. The graphs represent the mean values ± SD from at least three independent replicates. Statistical significance of the changes in cytokine concentration in comparison to control without implant was tested with the *t*-test: * *p* ≤ 0.005, ** *p* ≤ 0.001.

The cytotoxicity of silicone towards the cells in our 3D skin equivalent was assessed based on the activity of LDH released into the culture medium by damaged cells at days 5, 10, 15, 20, 25, and 30 after 3D skin equivalent production (Figure 5a). Implanting silicone into our 3D skin equivalents did not trigger LDH release (Figure 5a) but did induce a remarkable change in the overall immune response signified by statistically significant rises in concentrations of proinflammatory cytokines IL-6, IL-8, IL-33, and MCP-1, with the highest increases measured for IL-1α and TNF-α (Figure 5b–g).

## 4. Discussion

In vitro testing platforms for analysis of biocompatibility of materials implanted into the human body should strive to represent physiological conditions as closely as possible. 3D tissue equivalents are an advancement in this field, as they resemble the native tissue architecture more accurately than do mere 2D cell cultures [10,33].

Based on our experience in establishing a 3D pleura equivalent, which proved to be applicable for investigating pleural infections, we aimed to engineer a 3D skin equivalent [31,32]. To verify the aforementioned goal of close resemblance to human skin, we compared our 3D skin equivalent histologically to specimens of human skin and were able to demonstrate a skin-typical layered structure (Figure 2). Proteins characteristic to each stratum were expressed, proving the similarity of the tissue architecture of our in vitro model in further detail (Figure 3). The immunofluorescence staining showed similar distribution patterns of epithelial markers in the correct order although they were not identical to actual human skin.

Cell–cell contacts, specifically tight junctions, are essential to the skin’s barrier function. We were able to prove the presence of tight junctions in our 3D skin equivalent (Figure 4) and thus the presence of an intact barrier. Combined, these findings demonstrate that our in vitro 3D skin equivalent effectively reproduces important characteristics of the in vivo morphology of human skin with similar biophysical and biomolecular mechanisms.

Establishing this skin equivalent posed various challenges. In attempts to have the collagen matrix for the dermal structure be produced entirely by the contained fibroblasts, as other groups have demonstrated, the resulting thickness of the dermal layer was not satisfactory [34]. In our preliminary trials, we were not successful at using commercially available human collagen. The coculture either did not adhere to the transwell walls and collapsed in the lower collagen concentrations or showed constant shrinkage in higher concentrations. This highlights the influence of collagen stiffness as well as alignment and the orientation of collagen fibres, which was not further examined in this study. In order to achieve a stable, full thickness skin equivalent, we resorted to adding rat collagen [35]. Thus, our 3D skin equivalent is not entirely human-derived. Another way to overcome this hurdle is using decellularised dermis as a foundation for keratinocytes [23]. In this case, the dermal component is completely human, but it is obtained from actual human skin specimens. This limits availability and perhaps, more importantly, reproducibility since the influence of donor-individual factors such as age, medication, or disease cannot be eliminated. Our approach using only commercially available cells and components ensures the consistency and comparability of results.

It has to be acknowledged that our 3D skin equivalent still lacks components of the complex in vivo situation.

One major drawback of this and other current skin equivalents surely is the lack of immune cells, namely Langerhans cells of the epidermis, and a variety of specialised immune cells contained in the dermis [28]. Particularly when assessing an implant’s effect on tissues, the elicited immunologic response is of great interest. Nevertheless, our 3D skin equivalent is an appropriate platform to approach these questions since keratinocytes as our first barrier to the outside world play a major role in immune response [28]. We plan to advance our current 3D skin equivalent by coculturing immune cells as well, as has been done in models for inflammatory skin diseases [33].

As the models are kept static in an incubator under constant conditions, a further limitation of our 3D skin equivalent and basically all coculture models is the absence of physical influential factors such as movement, UV exposure, and pressure or temperature changes, which are present in vivo.

Recently, constructed 3D skin equivalents have been used to analyse the skin integration of percutaneous devices, demonstrating one of many potential applications for this type of tissue model [23,30]. While percutaneous devices are usually implanted only temporarily, a testing platform for materials intended to remain in the human body for prolonged periods of time, possibly a lifetime, seems highly interesting. Thus, we experimented with the integration of a foreign material into our 3D skin equivalent. To our knowledge, this is the first report of a 3D skin equivalent enhanced by an intradermal implant.

The crucial development of the characteristic skin strata (Figure 2 and Figure 3) and tight junctions (Figure 4) was not inhibited by the implantation of a foreign body. We did notice a thinning of the epidermal layer, while dermal thickness was not influenced. A possible reason for this observation is discussed in the next section.

Silicone seemed particularly fitting for this study for several reasons: It is a widely used material for implants, which are mostly intended to be kept inside the body for as long as possible, and due to its elasticity, it seemed well manageable for the first trials with foreign materials in our 3D skin model. Furthermore, the immunologic response of in vitro keratinocytes and fibroblasts to silicone has not been reported yet despite silicone implants often being inserted into areas with little subcutaneous tissue coverage such as digits and mammae [27].

Our LDH assay aligns with the reported cytocompatibility of silicone, as there was no statistical significance between cell death in samples of skin equivalents with and without such implants [14,27].

Keratinocytes as immunologically active barrier cells secrete various proinflammatory cytokines, among them being the cytokines we tested for in this study (IL-1 α, IL-6, IL-8, IL-33, MCP-1, and TNF- α) and with all of them showing statistically significant higher concentrations after addition of the silicone implant to our 3D skin equivalent [28,36,37]. Silicone has been shown to elevate the levels of monocyte secretion of IL-6 and TNF-α in vitro [27]. This intersects with our results of keratinocytes releasing statistically significantly more IL-6 and TNF-α, with TNF-α showing a particularly strong reaction in skin equivalents with implant.

MCP-1 mediates acute and chronic inflammation, is a strong attractant for macrophages, and plays an important role in contact dermatitis, where it is secreted by keratinocytes [38,39].

IL-8, as a chemoattractant for various immune cells, showed a doubling in concentration when keratinocytes were confronted with the silicone implant [40].

In acute inflammation, IL-33, as an alarmin, is expressed in human keratinocytes in contrast to murine cells [37,41]. The significant rise in IL-33 concentration in our 3D skin equivalents with implants is thus not only indicative of the proinflammatory potential of silicone but demonstrates once more the importance of research platforms based on human cells such as the one introduced here, as this species-specific reaction could not be replicated in a murine model.

Another interesting observation is the intensified keratin 17 expression in 3D skin equivalents with an implant compared to ones without an implant. Keratin 17, as a filament protein, is elevated in growth under pathological conditions and inflammation processes of skin, further matching the stress cytokine response [42,43].

An up to fourfold increase in the 3D skin equivalent’s cytokine response to the implanting of a foreign matter demonstrates that our 3D skin equivalent can be used as a platform for testing different materials for their inflammatory effect on skin.

Since the silicone sample was implanted before polymerisation of collagen prior to cultivation of the 3D skin equivalent, we exclude this completely atraumatic process as a possible cause for the observed stress response.

These proinflammatory conditions provoked by the silicone implant might be one reason for the epidermal layer being noticeably thinner in the implant model compared to the model without implant mentioned above. By contrast, our histological examinations demonstrated that the dermal growth patterns were unaffected by the silicone implant. It is possible that the observed cytokine response representing inflammatory activity slows down the differentiation and cornification of keratinocytes. Insufficient nourishment of cells can be ruled out as a possible cause for the epidermal thinning, as all epidermal cells in the 3D skin equivalents with implants presented this specific characteristic even in border areas, which were not in contact with the implant. Nevertheless, it has to be noted that in vivo implants are placed in mature tissues, which is in contrast to our model, where the implant is already present during the maturation process, possibly negatively influencing cell activities. Although silicone has been shown to affect fibroblast viability in 2D cultures, we could not observe a difference in fibroblast maturation [44]. On the other hand, silicone dressings are explicitly used to treat keloids and hypertrophic scars by reducing keratinocyte stimulation [45]. Although this effect is mostly attributed to hydration, it does align with our evident epidermal thinning.

While to our knowledge, this is the first description of immunohistochemical trials on a 3D skin equivalent with an intradermal implant and the first in vitro analysis of the influence of silicone on keratinocytes and fibroblasts, several limitations have to be considered.

We observed our 3D skin equivalents with implant for 30 days. Silicone implants are left in the body for years and decades, but we currently cannot make assumptions on the long-term effects based on our trials.

Since our model consists of epidermal and dermal equivalent only, we also cannot draw conclusions for implants embedded in the deep subcutaneous or submuscular plane without direct skin contact. It has to be noted however that no direct contact between keratinocytes and the silicone sample was needed for the described immunologic response.

Despite our efforts, we were not able to preserve the implant in our histological samples after the procedure of deparaffinisation of paraffin-embedded sections before staining. While this did not allow for optimal visual representation of our trials in the figures shown, it did not affect the results.

We see multiple future applications for this newly established 3D skin equivalent with an implant. A broad variety of other materials can be implanted and evaluated for their cytotoxic and proinflammatory properties. By inoculating bacteria, it could also be a useful tool for in vitro investigations on implant infections.

## 5. Conclusions

We presented a newly established, full-thickness, 3D skin equivalent with a dermal implant, which effectively reproduces the in vivo morphology of human skin and thus could be of value for future in vitro studies on the biocompatibility of various materials for implants.

Furthermore, this study is the first to demonstrate the noncytotoxic and proinflammatory effect of silicone on human keratinocytes and fibroblasts.

## Figures and Tables

**Table 1:** Immunofluorescence reagents.

	Source	Dilution
**Primary Antibody**		
Filaggrin	Abcam plc, Cambridge, UK	1:400
Involucrin	Abcam, UK	1:200
Cytokeratin 1	Abcam, UK	1:200
Keratin 17	Cell Signaling Technology, Danvers, MA, USA	1:400
E-Cadherin	Proteintech Europe, Manchester UK	1:250
ZO-1	Proteintech, UK	1:1000
**Secondary Antibodies**		
Goat anti-rabbit Alexa Fluor 488	Cell Signaling Technology, Danvers, MA, USA	1:1000
Goat anti-rabbit Alexa Fluor 555	Cell Signaling Technology, USA	1:1000
Goat anti-rabbit Alexa Fluor 647	Invitrogen, Themo Fisher Scientific, Waltham, MA, USA	1:1000

## Data Availability

The data presented in this study are openly available in Zenodo at https://doi.org/10.5281/zenodo.10309217 (accessed on 18 January 2024).

## Supplementary Materials

The following supporting information can be downloaded at: https://www.mdpi.com/article/10.3390/biomedicines12010224/s1, Figure S1: Comparison of the graphic abstract of our 3D skin equivalent and immunostaining of skin layer markers to further demonstrate the succesful stratification of our 3D skin equivalent.
