# Supplementary material for: A Platform for Testing the Biocompatibility of Implants: Silicone Induces a Proinflammatory Response in a 3D Skin Equivalent
Source: Biomedicines. 2024 Jan 19;12(1):224. doi: 10.3390/biomedicines12010224 (PMC10813245; doi:10.3390/biomedicines12010224)
Supplement: Supplementary file 1 [file biomedicines-12-00224-s001.zip › biomedicines-2790267-supplementary.pptx]

## Slide 1
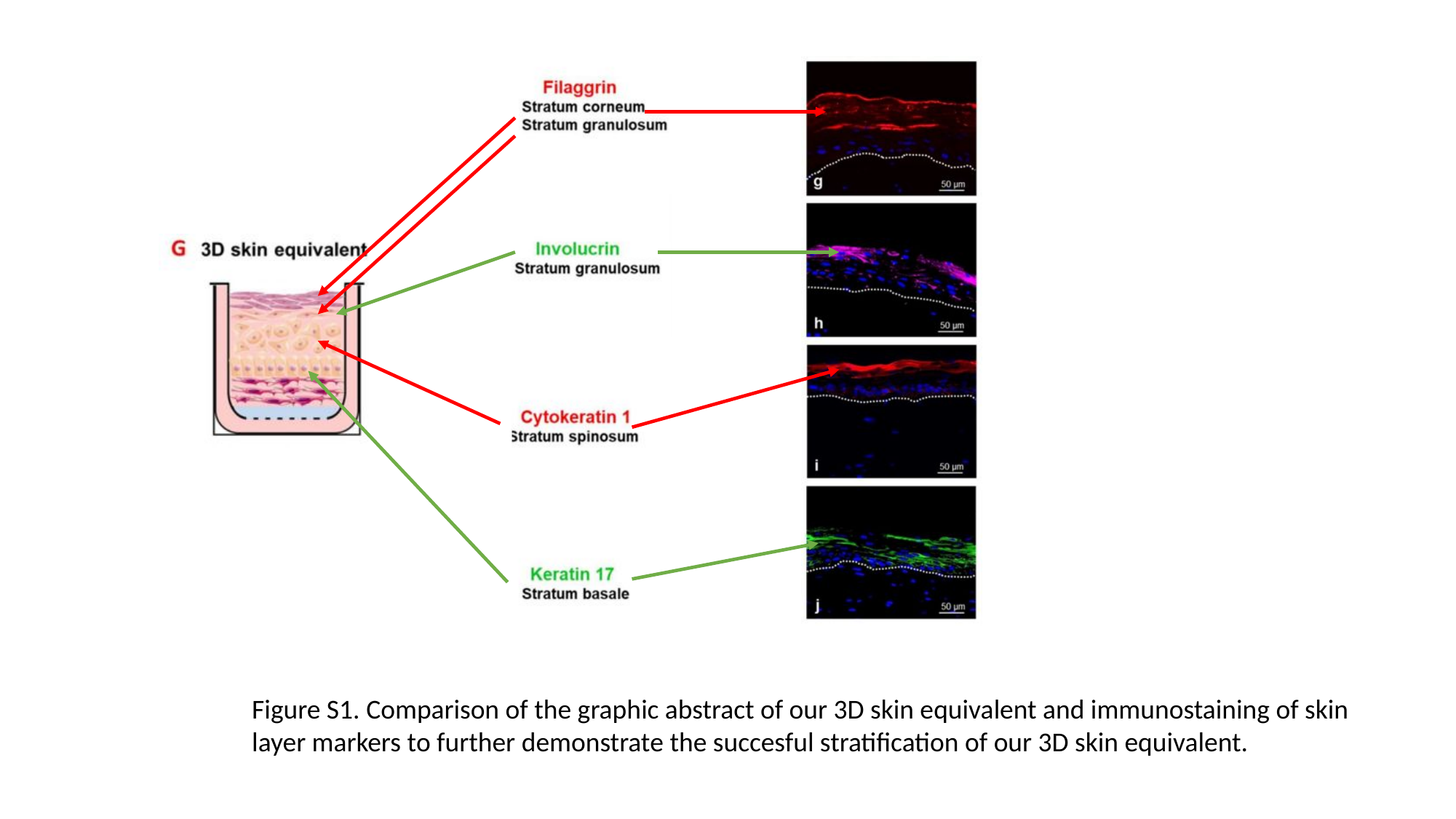

Figure S1. Comparison of the graphic abstract of our 3D skin equivalent and immunostaining of skin layer markers to further demonstrate the succesful stratification of our 3D skin equivalent.
